# Draft Genome Sequence of the Principal Etiological Agent of Farmer’s Lung Disease, *Saccharopolyspora rectivirgula*

**DOI:** 10.1128/genomeA.01340-14

**Published:** 2014-12-18

**Authors:** Coralie Barrera, Benoît Valot, Bénédicte Rognon, Christophe Zaugg, Michel Monod, Laurence Millon

**Affiliations:** a UMR 6249 Chrono-Environnement, University of Franche-Comté, Besançon, France; b Parasitology-Mycology Department, University Hospital of Besançon, Besançon, France; c Dermatology Department, University Hospital, Lausanne, Switzerland

## Abstract

*Saccharopolyspora rectivirgula* is the main cause of farmer’s lung disease. The development of recombinant antigens to standardize the serodiagnosis of the disease requires knowledge of the *S. rectivirgula* genome. We sequenced the genome of an environmental strain, *S. rectivirgula* DSM 43113. A total of 3,221 proteins were found to be encoded in a short 3.9-Mb genome.

## GENOME ANNOUNCEMENT

The thermophilic actinomycete *Saccharopolyspora rectivirgula* (syn., *Micropolyspora faeni, Faenia rectivirgula*) is the main etiological agent of farmer’s lung disease (FLD), a common type of occupational hypersensitivity pneumonitis (1). The FLD diagnosis is complex because it requires an association of clinical and biological markers, including serum-precipitating antibodies against offending antigens (2, 3). The serological tests that are routinely performed rely on unstandardized crude antigens (4). The working group of lung disease experts has stressed the need for standardized antigens for FLD diagnosis (5). The development of a recombinant antigen assay requires knowledge of the *S. rectivirgula* genome. We present here the partial sequence of the genome of *S. rectivirgula* strain DSM 43113 isolated from FLD-linked hay.

The conditions for growth and DNA extraction of *S. rectivirgula* were described previously (6). The whole genome was sequenced with the 454 FLX technology (454 Roche GS FLX; Roche, Mannheim, Germany). This led to 250,375 reads, for a total of 92,501,101 bp. *De novo* assembly was performed by GS *de novo* Assembler software (version 12.0.01.14) using the default settings. We obtained 50 contigs (>200 bp) for a total of 3,961,468 bp, and the *N*_50_ contig size was found to be 266,613 bp. The sequences were annotated with the NCBI Prokaryotic Genome Annotation Pipeline (http://www.ncbi.nlm.nih.gov/genome/annotation_prok/). A total of 3,221 proteins, 5 rRNAs, and 50 tRNAs were found.

The genome size and contents of this draft genome were highly similar to those of the strain *S. rectivirgula* type strain DSM 4374 (7). The comparison of their draft genomes with nucmer 3.1 (8) revealed 99.99% identity on 99.8% of their total lengths, with only 147 single-nucleotide polymorphisms (SNPs). The genome of strain DSM 43113 displayed 78 insertions, for a sum of 9,868 bp, compared to that of the strain DSM 4374. Conversely, the genome of DSM 4374 displayed 255 insertions, for a total of 12,567 bp, compared to that of DSM 43113. These data confirm that *S. rectivirgula* (3.9 Mb) has a smaller genome than those of the 2 other *Saccharopolyspora* species (*S. erythraea*, 8.21 Mb; *S. spinosa*, 8.58 Mb) (7).

These sequence data will facilitate the production of recombinant immunogenic proteins that could be further used as antigens for improved and standardized assay for FLD diagnosis (6).

### Nucleotide sequence accession numbers.

This whole-genome shotgun project has been deposited in DDBJ/EMBL/GenBank under the accession no. JNVU00000000. The version described in this paper is the first version, JNVU01000000.
